# Sterile Inflammation in *Drosophila*

**DOI:** 10.1155/2015/369286

**Published:** 2015-04-08

**Authors:** Zeeshan Shaukat, Dawei Liu, Stephen Gregory

**Affiliations:** School of Biological Sciences, University of Adelaide, North Terrace, Adelaide, SA 5006, Australia

## Abstract

The study of immune responses in *Drosophila* has already yielded significant results with impacts on our understanding of vertebrate immunity, such as the characterization of the Toll receptor. Several recent papers have focused on the humoral response to damage signals rather than pathogens, particularly damage signals from tumour-like tissues generated by loss of cell polarity or chromosomal instability. Both the triggers that generate this sterile inflammation and the systemic and local effects of it are only just beginning to be characterized in *Drosophila*. Here we review the molecular mechanisms that are known that give rise to the recruitment of *Drosophila* phagocytes, called hemocytes, as well as the signals, such as TNF*α*, that stimulated hemocytes emit at sites of perceived damage. The signalling consequences of inflammation, such as the activation of JNK, and the potential for modifying this response are also discussed.

## 1. Introduction

The inflammatory response to infection by pathogens has been intensively studied for many years both in humans and in all the major model organisms. More recently, there has been increasing interest in understanding the situations in which inflammation arises without an external pathogen [1]. These include almost any stimulus that gives tissue damage, such as burns, as well as autoimmune disease, atherosclerosis, stroke, and cancer. The molecular details of these self-induced inflammatory responses are now becoming clearer, though there appears to be a wide variety of triggers and outcomes that range from beneficial to lethal [2]. To make sense of the complexity and sort out causes from effects, model organisms amenable to genetic manipulation can be extremely useful. In this review we will focus on recent progress in understanding the causes and effects of sterile inflammation in* Drosophila*, which has many advantages for this kind of work.

The immune system in* Drosophila* is relatively simple: they lack adaptive immunity but have a robust innate immune system that has many functional and molecular similarities to that of vertebrates [3]. The immune cells in flies are collectively described as hemocytes; in normal animals they consist primarily of plasmatocytes with a phagocytic role as well as some crystal cells for melanization and clotting [4, 5]. The humoral innate immune response includes several antimicrobial peptides (AMPs), which can be produced from most epithelia, and upon infection are generated at high levels from the fat body, the equivalent of the vertebrate liver [6]. In addition there are a range of extracellular signalling molecules that are used to identify the presence of pathogens and trigger an inflammatory response [5]. These triggers include well known factors such as components of bacterial cell walls but also less well understood mechanisms such as an extracellular protease cleavage cascade that results in the activation of the IL-1R-like receptor Toll and the NF*κ*B pathway. Although the inflammatory response in* Drosophila* lacks several features of vertebrate inflammation, such as heat, redness, and extravasation of leucocytes, some signalling pathways regulating the response are conserved and indeed were discovered in* Drosophila*. In both insects and mammals there is the recruitment of immune cells to the affected site and the release of chemicals and peptides intended to damage pathogens; this is the process we are describing as inflammation.

It has become clear that, in the case of sterile inflammation, although there is no pathogen present, the inflammatory response is often similar to that seen in infection, and many of the same pathways are used [1, 5]. The inflammatory triggers, however, are not from a pathogen and must be generated by changes to normal cells that expose altered or mislocalized self-molecules to the immune system to generate a damage signal. These signals, known as damage-associated molecular patterns (DAMPs), are currently the subject of intensive research and may include extracellular chromatin, ATP, cytoskeletal molecules, and mitochondrial components [2]. In vertebrates, these are detected by diverse receptors including the many Toll-like receptors (Tlrs), but in flies the situation is likely to be less complex. In the next section we will examine the types of cellular damage that can give rise to sterile inflammation during larval life in* Drosophila* and the molecular triggers involved.

## 2. Sources of Sterile Inflammation Triggers in* Drosophila*

Many DAMPs are the normal molecules of the cytoplasm or nucleus that become immunogenic when exposed to extracellular environment. For example, in case of necrosis, nuclear or mitochondrial DNA is released into the extracellular environment and acts as a DAMP. Other DAMPs identified in vertebrates include high mobility group box 1 (HMGB1), reactive oxygen species (ROS), cytoskeletal molecules, nucleotides (e.g., ATP) and nucleosides (e.g., adenosine), uric acid, phosphatidylserine (PS), heat shock proteins (HSPs), hyaluronan, heparan, syndecan, and probably others which are still unidentified [2]. Some of them (e.g., nucleotides) are conserved between species and also shared by all types of tissue injuries [7].

### 2.1. Necrotic Cells

Necrosis is the main source of damage signals in many tissue injuries such as tumours, thermal effects, mechanical trauma, ischemia, hypoxia, and apoptosis-mutants. Acidification and the oxidative environment of necrotic cells are thought to cause proinflammatory changes to DAMPs inside and out of the cell. For example, high mobility group box-1 (HMGB1) is a nonhistone, DNA binding protein that has been implicated as a DAMP in vertebrates [2, 8]. As a result of ROS, partially oxidised HMGB1 is released out of the necrotic cells and binds to extracellular mediators of inflammation (such as ssDNA or lipopolysaccharides) and promotes activation of Toll-like receptors [9]. This mechanism has not been studied in detail in* Drosophila*, but we have found that loss of HMGB1 reduces sterile inflammation (our unpublished results). The release of DNA from necrotic cells may also contribute to a conserved inflammatory response, as* Drosophila* mutants that block DNAseII function show a humoral response [10] with similarities to vertebrate signalling [11].

Reactive oxygen species are also released from necrotic cells in* Drosophila* and act as an immediate damage signal which may trigger the recruitment of hemocytes to the injured tissue [12–14]. ROS and TNF*α* (Eiger) released from necrotic neuronal cells can trigger JNK activation in surrounding cells [12]. The activated JNK pathway triggers apoptosis, hemocyte recruitment, and wound healing [12, 15]. This occurs at least partly by activation of matrix metalloproteinases (MMPs) which can result in the production of DAMPs by digesting basement membrane [16, 17], though their primary function is one of repair [18]. TNF*α*, phosphatidylserine (PS), and other DAMPs have been shown to enhance the activation of the prophenoloxidase activating system (PAS) at the site of injury. Activation of the PAS melanizes wound clots and other encapsulated tissues or pathogens [19], as well as triggering a systemic response [20].

### 2.2. Undead Cells

Undead cells, such as cells that fail to apoptose due to caspase mutations [21], are known to promote the activation of the extracellular protease Persephone as a trigger of the innate immune response [22, 23]. Persephone acts as a sensor in the hemolymph, which informs the insect about the presence of stress, damage, or pathogens [24]. The trigger for Persephone in sterile inflammation is not known, but we speculate that the release of necrotic material (e.g., intracellular proteases) can trigger activation of Persephone and the cleavage cascade that produces a systemic immune response [3, 5, 6, 22]. For example, it is known that some soluble DAMP in the hemolymph is required for the systemic immune response seen in apoptosis mutants [22]. Restraining the systemic activation of the immune proteases are serpins such as Necrotic, which is expressed ubiquitously and helps in establishing a localized signal gradient at the site of damage by damping the overall proteolytic activity in the body. This localization of the signal assists the recruitment of hemocytes [19].

### 2.3. Wounds

Wounding, in a nonpathogenic environment, promotes a similar activation of the immune response as described above, because wounds contain both necrotic and stressed cells. Sterile wounding in* Drosophila* is thought to stimulate the pathogen response as a protective measure against expected infection [25, 26], though, at least in adults, the intensity of the response may be less than that for an infection [27]. In sterile wounding, release of DAMPs at the wound site has been proposed to result in activation of Persephone and differentiation of lamellocytes from precursor hemocytes [4, 5, 24, 28]. Lamellocytes are involved in the encapsulation of target tissue (normally degenerating tissues and oversized pathogenic invaders) and then in melanizing them via activation of the phenoloxidase cascade. Basement membrane (BM) disruption acts as a trigger for the immune response in wound regions as well as metastasizing tumors [16, 29, 30]. Laminin is a major component of the BM which acts as a checkpoint for self/nonself and normal/damaged tissue. It acts as an inhibitory ligand for hemocytes [17, 31] and is also important for cell integrity. Cell integrity (cell-cell adhesion and apicobasal polarity) of self-tissues also acts as a determinant for the immune response. Loss of both BM and cell integrity is required to target an otherwise self-tissue for encapsulation by lamellocytes [17].

### 2.4. Tumors

Tumor interactions with the immune system are typically required for their growth and metastasis [4]. Tumour growth generates signals that have been linked to hemocyte proliferation and recruitment [32–34]. In* Drosophila*, tumor cells activate TNF*α*, Pvf/Pvr, and the Toll pathway to trigger the systemic immune response (see below). The loss of apicobasal cell polarity often seen in malignant outgrowth also induces recruitment of hemocytes and encapsulation [33]. Triggering mechanisms have not been explored in detail, but expression of an oncogene (Ras^V12^) in* Drosophila* showed hyperplastic growth and increased expression of metalloproteinases [34, 35]. This is relevant because increased expression of MMPs causes degradation of basement membrane, which leads to inflammation [16, 34]. In addition, exposure of phosphatidylserine on the surface of Ras^V12^ mutant cells [34] can trigger the prophenoloxidase activating system which gives melanisation and encapsulation [19].

Finally, cancer cells often exhibit a high rate of genetic change due to chromosomal instability (CIN). CIN can provide variability and adaptability but at the cost of generating ROS and cellular stress which often results in cell death [36, 37]. Release of cellular debris from CIN tissue gives both localized and systemic activation of the Toll pathway (our unpublished data). CIN also leads to DNA damage [36–38]. Unrepaired DNA damage in* Drosophila* elicits an innate immune response which leads to systemic activation of JAK/STAT signaling, hemocyte proliferation, and melanization [16, 39, 40].

## 3. Effects of the Inflammatory Response

Inflammation in* Drosophila* typically results in the production of antimicrobial peptides and the recruitment of hemocytes [4, 5, 41]. Antimicrobial peptides are not known to have strong effects on the organism in the absence of a pathogen, though they can potentially affect neural tissue [42] and promote autoimmunity [43]. Recruitment of hemocytes, on the other hand, has profound implications for the tissue involved as well as for the animal as a whole.

**Figure 1:**
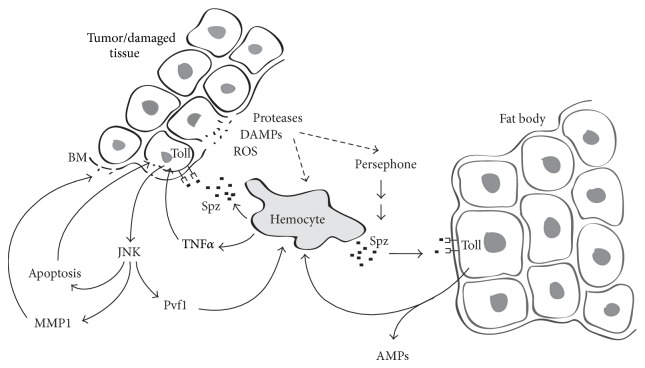
Tumors appear as damaged tissue, releasing DAMPS including ROS and triggers that recruit hemocytes and activate the proteolytic cascade that results in the production of active Spaetzle (Spz). Spz acts both locally and in the fat body to stimulate signalling through Toll/NF*κ*B. Hemocytes also release the short-range signal TNF*α* which, along with Toll signalling, activates JNK in the target tissue. JNK signalling produces cytokines like Pvf1, degrades basement membrane via matrix metalloproteases (MMP1), and promotes apoptosis. All these effects tend to recruit and activate further hemocytes at the damage site to generate an effective inflammatory response.

A population of hemocytes constantly circulates in the haemolymph, having access to the basal surface of most organs and tissues. As described above, there are a number of signals released by damaged or aberrant tissues that lead to the accumulation of hemocytes at the site, in a process that is thought to involve capture of passing hemocytes rather than active migration, at least in the larva [29]. In a sterile wound, both plasmatocytes and crystal cells gather, degranulating to release clotting factors as well as a range of signalling molecules [5]. These signals include the TNF*α* homolog Eiger and the cytokines Unpaired-3 and Spaetzle (Figure 1), showing clear similarity to vertebrate sterile inflammation [2].

### 3.1. Cytokine Signaling

The production of the NGF*β* homolog Spaetzle [44] by hemocytes is primarily thought to drive systemic rather than local immune responses in* Drosophila*. For example, activation of the Spaetzle receptor Toll just in hemocytes does not improve immune responses [45]. Instead, the principal immune effect of the Spaetzle signal is seen in the fat body [33], equivalent to the vertebrate liver, which responds by becoming the primary source of antimicrobial peptides [46, 47]. Recent work has shown, in response to tissue dysplasia, that Spaetzle activating Toll in the fat body is also needed to drive TNF*α* mediated cell death in the aberrant tissue [33]. Spaetzle is a highly regulated signal, being secreted as a proprotein that requires protease cleavage in order to be active. A wide range of extracellular proteases that are either produced by bacteria or activated by bacterial molecules are known to generate active cleaved Spaetzle during infections [5]. In sterile inflammation, activation of the protease Persephone is probably required [22, 23], though how it is regulated is not known. We speculate that the same necrotic cell death that attracts hemocytes can release normally intracellular proteases that trigger Persephone and the cleavage cascade that produces active Spaetzle. The molecular pathway by which the Spaetzle receptor Toll activates the humoral immune response has been analysed in detail and closely parallels the vertebrate pathway [3, 5, 6]. Still relatively unknown, however, are the transcriptional outputs of this pathway in response to DAMPs, beyond a handful of antimicrobial peptides. We do not know, for example, what targets of NF*κ*B might be relevant for the fat body and Toll-dependent death of tumour tissue [33]. Presumably this is mediated by the fat body signalling to increase the release of TNF*α* on the tumour by hemocytes, but the molecules used are not known. Spaetzle is also implicated in cell competition, where it activates signalling via Toll-like receptors to kill relatively unfit cells [48]. The source of Spz and the involvement of hemocytes in this process have not yet been determined.

Hemocytes also release the IL-6 related cytokine Unpaired-3, which is produced in a feedback response to Unpaired signalling from wounds or tumours [16]. Damaged tissue activates the JNK pathway which increases the transcription of Unpaired, Unpaired-2, and Unpaired-3, which are secreted by the tissue to activate JAK/STAT signalling in hemocytes that have been recruited, as well as from the fat body. JAK/STAT signalling produces more hemocyte secretion of the Unpaired cytokines in a positive feedback loop as well as driving hemocyte proliferation and lamellocyte differentiation [49]. This system resembles a simplified version of the mammalian use of interleukins and JAK/STAT signalling in inflammatory responses [50].

### 3.2. Tumor Necrosis Factor Signalling

While Spaetzle and Unpaired have systemic effects on hemocyte numbers, the primary effector molecule secreted by hemocytes in sterile inflammation is TNF*α* (Eiger) [51, 52]. TNF*α* is clearly secreted by hemocytes that have been recruited to sites of cellular damage [33] and possibly also by unstimulated hemocytes [53, 54]. TNF*α* signalling through the TNF receptor Wengen has two well described effects: activation of JNK signalling and cell death [52]. Strong and persistent activation of JNK leads to increased transcription of the proapoptotic genes* hid* and* reaper* [55], which TNF*α* also activates by a parallel pathway involving the TRIP homolog Nopo [56]. Consequently, cell death is a significant feature of normal inflammatory responses in* Drosophila* larvae. However, it is important to bear in mind that JNK also has many other functions [57], so, for example, if its apoptotic role is blocked, Eiger-JNK signaling can contribute to proliferation and metastasis [58–60]. Furthermore, JNK signalling can be protective in neural tissue, and this ROS-mediated protection by JNK activation is needed to survive even sterile wounds [20].

It is interesting to consider what constitutes the targeting signal of the innate immune response. Hemocytes, as the primary detectors of damage or pathogens, release active Spaetzle to give systemic Toll activation and Unpaired to drive hemocyte proliferation, but this does not explain how the response is focused on the site of infection/damage [53]. It appears that hemocyte recruitment and retention is essential to localize the response. Reacting to the as-yet poorly defined damage signals, hemocytes potentially reinforce their localization by generating more local damage. The release of TNF*α* causes cell death as well as JNK activation, which drives the secretion of basement membrane proteases such as MMP1, which is sufficient to generate hemocyte-localizing damage, as described above. Furthermore, we have found that local activation of Toll in the target tissue is essential for the normal apoptotic response. In this case, signalling through Toll/NF*κ*B in defective tissue activates JNK to produce MMP1 and recruit hemocytes (our unpublished data). This constitutes a local amplification loop perhaps resembling the vertebrate Toll-like receptor p75 that drives both NF*κ*B and JNK [52].

### 3.3. Reactive Oxygen Species

The role of reactive oxygen species in sterile inflammation is still unclear, though undoubtedly significant. Several ROS molecules have been implicated in antibacterial responses [3, 61] and in these cases they are typically generated to damage pathogens but also to stimulate hemocytes to generate a systemic response [62]. Less is known about how ROS might act in sterile inflammation: they can be produced by TNF*α* signalling and contribute to the resulting cell death [63], and they act with or without growth signals to activate JNK signalling in different tissues [20, 64]. The hydrogen peroxide produced by damaged tissue is necessary, at least in* Drosophila* embryos and zebrafish, for the recruitment of hemocytes or leukocytes [14, 65]. These studies implicate the calcium flash from wounding in triggering activation of the peroxide-generating enzyme DUOX to generate the ROS signal for attracting hemocytes. However, metabolic disruption in tumours or chromosomal instability also produces ROS [36, 66], so the same ROS signal may also be used in the absence of external wounding. As a damage signal, hydrogen peroxide has many advantages: it is readily produced by defective cells, it diffuses through membranes, and it is reactive enough to limit its own diffusion to a few cell diameters [67]. Following the recruitment of hemocytes to an inflammatory site, we expect that the production of TNF*α* by the hemocytes [33] increases ROS production in nearby cells [63, 68], as yet another positive feedback loop to encourage the death of damaged cells.

ROS production is strongly affected by a range of metabolic controls that are altered by inflammation [3]. In* Drosophila* this may be mediated by the fat body, which responds to necrosis by activating JNK targets such as FOXO that both increase antioxidant production and drive lipolysis, the normal response to starvation [21]. This energy wasting effect is commonly seen in both infections and cancer in all organisms, leading to cachexia that has been associated with elevated TNF levels and may respond to anti-inflammatory therapy [69].

## 4. Implications of Sterile Inflammation Control

It is perhaps surprising that the inflammatory response that is used to clear bacterial infections and to clot wounds should cause hemocytes to secrete TNF*α*, which damages the host more than the pathogen. Unwanted TNF*α* can be responsible for debilitating disease, as seen in allergies and autoimmune diseases that respond strongly to anti-TNF*α* therapies. Nonetheless, TNF*α* is a valuable protective mechanism, as TNF*α* inhibitor therapy in humans is associated with increased risk of infections and, significantly, cancer [70]. Experiments in* Drosophila* have underlined the need for localised TNF*α* production by hemocytes to control the growth of neoplastic tissue [16, 33]. In this context it is worth noting that loss of neutrophils, which share some features with hemocytes, is still an extremely common but obviously undesirable side effect of the front-line human chemotherapies [71]. Not only does neutropenia leave the patient vulnerable to infection, but also reduces the body's innate immune response to cancer.

While T-cell based immunotherapies are now available [72], effective cancer treatments using innate immune responses have not been developed. This may be partly because tumours must have typically developed some resistance to the innate immune response to have survived and grown to a point where they can be detected [7]. At this point the tumour may well be dependent on proinflammatory signalling, so therapies have been developed instead to combat inflammatory signalling. This stage of tumour development has been modelled in* Drosophila* by expression of active Ras, which can be used to generate tumours that depend on TNF*α* for invasive outgrowth [54]. These results underline the key role of JNK in modulating outcomes: the innate immune response can activate JNK signalling to kill damaged or infected tissue, but, in cases where cell death is blocked, the same signal promotes outgrowth and proliferation [55, 57, 60]. Clearly caution is needed in any intervention that alters the level of inflammatory response in either direction.

As this review has indicated, there is still a great deal that is unknown about the mechanisms that regulate sterile inflammation. Many inflammatory triggers from damaged tissue are yet to be characterized, particularly in model organisms. Similarly, we know little about the mechanisms that damp the many positive feedback systems to prevent a life-threatening excessive response to tissue damage. However, the signalling pathways and cytokines that mediate inflammation are now becoming relatively well studied and amenable to analysis by the mutagenesis screening approaches that have made* Drosophila* such a valuable tool [73]. With the current intense activity in the field we expect significant improvements in our understanding of sterile inflammation in the near future.

## References

[1] Rock K. L., Latz E., Ontiveros F., Kono H. (2010). The sterile inflammatory response. *Annual Review of Immunology*.

[2] Kono H., Onda A., Yanagida T. (2014). Molecular determinants of sterile inflammation. *Current Opinion in Immunology*.

[3] Buchon N., Silverman N., Cherry S. (2014). Immunity in *Drosophila melanogaster*—from microbial recognition to whole-organism physiology. *Nature Reviews Immunology*.

[4] Wang L., Kounatidis I., Ligoxygakis P. (2014). *Drosophila* as a model to study the role of blood cells in inflammation, innate immunity and cancer. *Frontiers in Cellular and Infection Microbiology*.

[5] Krautz R., Arefin B., Theopold U. (2014). Damage signals in the insect immune response. *Frontiers in Plant Science*.

[6] Ferrandon D., Imler J.-L., Hetru C., Hoffmann J. A. (2007). The *Drosophila* systemic immune response: sensing and signalling during bacterial and fungal infections. *Nature Reviews Immunology*.

[7] Lotfi R., Schrezenmeier H., Lotze M. T. (2009). Immunotherapy for cancer: promoting innate immunity. *Frontiers in Bioscience*.

[8] Lugrin J., Rosenblatt-Velin N., Parapanov R., Liaudet L. (2014). The role of oxidative stress during inflammatory processes. *Biological Chemistry*.

[9] Tsung A., Tohme S., Billiar T. R. (2014). High-mobility group box-1 in sterile inflammation. *Journal of Internal Medicine*.

[10] Mukae N., Yokoyama H., Yokokura T., Sakoyama Y., Nagata S. (2002). Activation of the innate immunity in *Drosophila* by endogenous chromosomal DNA that escaped apoptotic degradation. *Genes and Development*.

[11] Liu X., Sano T., Guan Y., Nagata S., Hoffmann J. A., Fukuyama H. (2012). *Drosophila* EYA regulates the immune response against DNA through an evolutionarily conserved threonine phosphatase motif. *PLoS ONE*.

[12] Yang Y., Hou L., Li Y., Ni J., Liu L. (2013). Neuronal necrosis and spreading death in a *Drosophila* genetic model. *Cell Death and Disease*.

[13] Moreira S., Stramer B., Evans I., Wood W., Martin P. (2010). Prioritization of competing damage and developmental signals by migrating macrophages in the *Drosophila* embryo. *Current Biology*.

[14] Razzell W., Evans I. R., Martin P., Wood W. (2013). Calcium flashes orchestrate the wound inflammatory response through duox activation and hydrogen peroxide release. *Current Biology*.

[15] Wu H., Wang M. C., Bohmann D. (2009). JNK protects *Drosophila* from oxidative stress by trancriptionally activating autophagy. *Mechanisms of Development*.

[16] Pastor-Pareja J. C., Wu M., Xu T. (2008). An innate immune response of blood cells to tumors and tissue damage in Drosophila. *Disease Models and Mechanisms*.

[17] Kim M. J., Choe K., Schneider D. S. (2014). Basement membrane and cell integrity of self-tissues in maintaining *Drosophila* immunological tolerance. *PLoS Genetics*.

[18] Stevens L. J., Page-McCaw A. (2012). A secreted MMP is required for reepithelialization during wound healing. *Molecular Biology of the Cell*.

[19] Bidla G., Hauling T., Dushay M. S., Theopold U. (2009). Activation of insect phenoloxidase after injury: endogenous versus foreign elicitors. *Journal of Innate Immunity*.

[20] Nam H.-J., Jang I.-H., You H., Lee K.-A., Lee W.-J. (2012). Genetic evidence of a redox-dependent systemic wound response via Hayan protease-phenoloxidase system in *Drosophila*. *The EMBO Journal*.

[21] Obata F., Kuranaga E., Tomioka K. (2014). Necrosis-driven systemic immune response alters SAM metabolism through the FOXO-GNMT axis. *Cell Reports*.

[22] Ming M., Obata F., Kuranaga E., Miura M. (2014). Persephone/Spätzle pathogen sensors mediate the activation of toll receptor signaling in response to endogenous danger signals in apoptosis-deficient *Drosophila*. *The Journal of Biological Chemistry*.

[23] El Chamy L., Leclerc V., Caldelari I., Reichhart J.-M. (2008). Sensing of ‘danger signals’ and pathogen-associated molecular patterns defines binary signaling pathways ‘upstream’ of Toll. *Nature Immunology*.

[24] Gottar M., Gobert V., Matskevich A. A. (2006). Dual detection of fungal infections in *Drosophila* via recognition of glucans and sensing of virulence factors. *Cell*.

[25] Stramer B., Winfield M., Shaw T., Millard T. H., Woolner S., Martin P. (2008). Gene induction following wounding of wild-type versus macrophage-deficient *Drosophila* embryos. *EMBO Reports*.

[26] Hyrsl P., Dobes P., Wang Z., Hauling T., Wilhelmsson C., Theopold U. (2011). Clotting factors and eicosanoids protect against nematode infections. *Journal of Innate Immunity*.

[27] Clark R. I., Woodcock K. J., Geissmann F., Trouillet C., Dionne M. S. (2011). Multiple TGF-*β* superfamily signals modulate the adult *Drosophila* immune response. *Current Biology*.

[28] Márkus R., Kurucz É., Rus F., Andó I. (2005). Sterile wounding is a minimal and sufficient trigger for a cellular immune response in *Drosophila melanogaster*. *Immunology Letters*.

[29] Babcock D. T., Brock A. R., Fish G. S. (2008). Circulating blood cells function as a surveillance system for damaged tissue in *Drosophila* larvae. *Proceedings of the National Academy of Sciences of the United States of America*.

[30] Rizki T. M., Rizki R. M. (1980). Developmental analysis of a temperature-sensitive melanotic tumor mutant in *Drosophila melanogaster*. *Wilhelm Roux's Archives of Developmental Biology*.

[31] Yurchenco P. D. (2011). Basement membranes: cell scaffoldings and signaling platforms. *Cold Spring Harbor Perspectives in Biology*.

[32] Wood W., Faria C., Jacinto A. (2006). Distinct mechanisms regulate hemocyte chemotaxis during development and wound healing in *Drosophila melanogaster*. *The Journal of Cell Biology*.

[33] Parisi F., Stefanatos R. K., Strathdee K., Yu Y., Vidal M. (2014). Transformed epithelia trigger non-tissue-autonomous tumor suppressor response by adipocytes via activation of toll and eiger/TNF signaling. *Cell Reports*.

[34] Hauling T., Krautz R., Markus R., Volkenhoff A., Kucerova L., Theopold U. (2014). A *Drosophila* immune response against Ras-induced overgrowth. *Biology Open*.

[35] Brumby A. M., Richardson H. E. (2003). *scribble* mutants cooperate with oncogenic Ras or Notch to cause neoplastic overgrowth in *Drosophila*. *EMBO Journal*.

[36] Shaukat Z., Liu D., Choo A. (2014). Chromosomal instability causes sensitivity to metabolic stress. *Oncogene*.

[37] Shaukat Z., Wong H. W. S., Nicolson S., Saint R. B., Gregory S. L. (2012). A screen for selective killing of cells with chromosomal instability induced by a spindle checkpoint defect. *PLoS ONE*.

[38] Wong H. W.-S., Shaukat Z., Wang J., Saint R., Gregory S. L. (2014). JNK signaling is needed to tolerate chromosomal instability. *Cell Cycle*.

[39] Ermolaeva M. A., Segref A., Dakhovnik A. (2013). DNA damage in germ cells induces an innate immune response that triggers systemic stress resistance. *Nature*.

[40] Karpac J., Younger A., Jasper H. (2011). Dynamic coordination of innate immune signaling and insulin signaling regulates systemic responses to localized DNA damage. *Developmental Cell*.

[41] Ganesan S., Aggarwal K., Paquette N., Silverman N. (2011). Nf-*κ*B/Rel proteins and the humoral immune responses of *Drosophila melanogaster*. *Current Topics in Microbiology and Immunology*.

[42] Cao Y., Chtarbanova S., Petersen A. J., Ganetzky B. (2013). Dnr1 mutations cause neurodegeneration in *Drosophila* by activating the innate immune response in the brain. *Proceedings of the National Academy of Sciences of the United States of America*.

[43] Gilliet M., Lande R. (2008). Antimicrobial peptides and self-DNA in autoimmune skin inflammation. *Current Opinion in Immunology*.

[44] Hepburn L., Prajsnar T. K., Klapholz C. (2014). A Spaetzle-like role for nerve growth factor *β* in vertebrate immunity to Staphylococcus aureus. *Science*.

[45] Schmid M. R., Anderl I., Vesala L. (2014). Control of *Drosophila* blood cell activation via Toll signaling in the fat body. *PLoS ONE*.

[46] Lindsay S. A., Wasserman S. A. (2014). Conventional and non-conventional *Drosophila* Toll signaling. *Developmental and Comparative Immunology*.

[47] Kounatidis I., Ligoxygakis P. (2012). *Drosophila* as a model system to unravel the layers of innate immunity to infection. *Open Biology*.

[48] Meyer S. N., Amoyel M., Bergantinos C. (2014). An ancient defense system eliminates unfit cells from developing tissues during cell competition. *Science*.

[49] Myllymäki H., Rämet M. (2014). JAK/STAT pathway in *Drosophila* immunity. *Scandinavian Journal of Immunology*.

[50] Stark G. R., Darnell J. E. (2012). The JAK-STAT pathway at twenty. *Immunity*.

[51] Bangi E. (2013). *Drosophila* at the intersection of infection, inflammation, and cancer. *Frontiers in Cellular and Infection Microbiology*.

[52] Igaki T., Miura M. (2014). The *Drosophila* TNF ortholog Eiger: emerging physiological roles and evolution of the TNF system. *Seminars in Immunology*.

[53] Pastor-Pareja J. C., Xu T. (2013). Dissecting social cell biology and tumors using *Drosophila* genetics. *Annual Review of Genetics*.

[54] Cordero J. B., Macagno J. P., Stefanatos R. K., Strathdee K. E., Cagan R. L., Vidal M. (2010). Oncogenic ras diverts a host TNF tumor suppressor activity into tumor promoter. *Developmental Cell*.

[55] Martín F. A., Peréz-Garijo A., Morata G. (2009). Apoptosis in *Drosophila*: compensatory proliferation and undead cells. *International Journal of Developmental Biology*.

[56] Ma X., Huang J., Yang L., Yang Y., Li W., Xue L. (2012). NOPO modulates Egr-induced JNK-independent cell death in *Drosophila*. *Cell Research*.

[57] Shaukat Z., Liu D., Hussain R., Khan M., Gregory S. L. The role of JNK signaling in responses to oxidative DNA damage.

[58] Brumby A. M., Goulding K. R., Schlosser T. (2011). Identification of novel Ras-cooperating oncogenes in *Drosophila melanogaster*: a RhoGEF/Rho-family/JNK pathway is a central driver of tumorigenesis. *Genetics*.

[59] Fan Y., Wang S., Hernandez J. (2014). Genetic models of apoptosis-induced proliferation decipher activation of JNK and identify a requirement of EGFR signaling for tissue regenerative responses in *Drosophila*. *PLoS Genetics*.

[60] Dekanty A., Barrio L., Muzzopappa M., Auer H., Milán M. (2012). Aneuploidy-induced delaminating cells drive tumorigenesis in *Drosophila* epithelia. *Proceedings of the National Academy of Sciences of the United States of America*.

[61] Kim S.-H., Lee W.-J. (2014). Role of DUOX in gut inflammation: lessons from *Drosophila* model of gut-microbiota interactions. *Frontiers in Cellular and Infection Microbiology*.

[62] Wu S.-C., Liao C.-W., Pan R.-L., Juang J.-L. (2012). Infection-induced intestinal oxidative stress triggers organ-to-organ immunological communication in *Drosophila*. *Cell Host & Microbe*.

[63] Kanda H., Igaki T., Okano H., Miura M. (2011). Conserved metabolic energy production pathways govern Eiger/TNF-induced nonapoptotic cell death. *Proceedings of the National Academy of Sciences of the United States of America*.

[64] Ohsawa S., Sato Y., Enomoto M., Nakamura M., Betsumiya A., Igaki T. (2012). Mitochondrial defect drives non-autonomous tumour progression through Hippo signalling in *Drosophila*. *Nature*.

[65] Niethammer P., Grabher C., Look A. T., Mitchison T. J. (2009). A tissue-scale gradient of hydrogen peroxide mediates rapid wound detection in zebrafish. *Nature*.

[66] Pfau S. J., Amon A. (2012). Chromosomal instability and aneuploidy in cancer: from yeast to man. *The EMBO Reports*.

[67] Winterbourn C. C. (2008). Reconciling the chemistry and biology of reactive oxygen species. *Nature Chemical Biology*.

[68] Morgan M. J., Liu Z.-G. (2010). Reactive oxygen species in TNF*α*-induced signaling and cell death. *Molecules and Cells*.

[69] Argilés J. M., López-Soriano F. J., Busquets S. (2012). Counteracting inflammation: a promising therapy in cachexia. *Critical Reviews in Oncogenesis*.

[70] Targownik L. E., Bernstein C. N. (2013). Infectious and malignant complications of TNF inhibitor therapy in IBD. *American Journal of Gastroenterology*.

[71] Dinan M. A., Hirsch B. R., Lyman G. H. (2015). Management of chemotherapy-induced neutropenia: measuring quality, cost, and value. *Journal of the National Comprehensive Cancer Network*.

[72] Mellman I., Coukos G., Dranoff G. (2011). Cancer immunotherapy comes of age. *Nature*.

[73] St Johnston D. (2002). The art and design of genetic screens: *Drosophila* melanogaster. *Nature Reviews Genetics*.

